# Modulation of CAT-2B-Mediated l-Arginine Uptake and Nitric Oxide Biosynthesis in HCT116 Cell Line Through Biological Activity of 4′-Geranyloxyferulic Acid Extract from Quinoa Seeds

**DOI:** 10.3390/ijms20133262

**Published:** 2019-07-02

**Authors:** Sara Franceschelli, Daniela Maria Pia Gatta, Mirko Pesce, Alessio Ferrone, José Luis Quiles, Salvatore Genovese, Francesco Epifano, Serena Fiorito, Vito Alessandro Taddeo, Antonia Patruno, Alfredo Grilli, Mario Felaco, Lorenza Speranza

**Affiliations:** 1 Department of Psychological, Health and Territorial Sciences, University G. D’Annunzio, 66100 Chieti, Italy; 2 Department of Medicine and Science of Aging, University G. D’Annunzio, 66100 Chieti, Italy; 3 Department of Physiology, Institute of Nutrition and Food Technology José Mataix, Biomedical Research Centre, University of Granada, 18071 Granada, Spain; 4 Department of Pharmacy, University Gabriele D’Annunzio of Chieti-Pescara, Via dei Vestini 31, 66100 Chieti Scalo (CH), Italy

**Keywords:** nitric oxide, l-arginine, oxidative stress, CAT-2B, inflammation, IL-6, TNF-α

## Abstract

*Chenopodium quinoa Wild* is a “pseudocereal” grain which attracts a lot of attention in the scientific community as it has a positive effect on health. Here, we investigate the presence of biologically active O-prenylated phenylpropanoids in the ethanol extract of commercially available quinoa seeds. We claim that 4′-Geranyloxyferulic acid (GOFA) was the only phytochemical product found that belongs to quinoa’s group secondary metabolites. We studied the changes in the oxidative and inflammatory status of the cellular environment in HCT 116 cell line processed with quinoa extract and its component GOFA; the implementation was done through the analysis of the antioxidant enzymes (SOD and CAT), the pro-inflammatory components (iNOS, IL-6 and TNF-α), and the products of intermediary metabolism (ONOO^−^, O_2_^−^). Moreover, the l-arginine uptake was proposed as a target of the tested compounds. We demonstrated that the GOFA, through a decrease of the CAT-2B expression, leads to a reduction of the l-arginine uptake, downregulating the harmful iNOS and restoring the altered redox state. These results propose a new molecular target involved in the reduction of the critical inflammatory process responsible for the cancer progression.

## 1. Introduction

The control of colorectal cancer (CRC) can be implemented by the potential of alternative therapies. In the past few years, plant extracts, which have anti-inflammatory, antioxidant, anti-proliferative, and anti-angiogenic properties, and are thus able to block, reverse, or prevent tumor growth, have been used as anticancer therapy [1]. The potential of nutraceutical compounds in reducing cancer progression has been largely explored. In fact, modern scientific researches have evidenced that a vast variety of medicinal aspects of plant extracts can be referred to the components, which can have a successful anticancer effect [2].

Chronic inflammation, accompanied by an imbalance of the cellular redox state, has been implicated as a potential biological mechanism in the progression of colorectal cancer in humans. This condition leads to the release of inflammatory cytokines and chemokines which promote proliferation, angiogenesis, invasion, and metastasis, and facilitate tumor growth [3]. Moreover, inflammation is related to the formation of reactive oxygen and nitrogen species (ROS/RNS) which have been suggested as a mechanism underlying the pathophysiology of CRC [4]. In fact, the phlogistic mediators NO, and the pro-inflammatory cytokine TNF-alpha, can be considered as key mediators of inflammatory processes which activate angiogenic processes and determine the synthesis of different chemokines in the colonic mucosa of CRC patients [5,6].

Arginine is implicated in several biosynthetic pathways which significantly influence cancer cell proliferation and tumor biology. Arginine traverses the cell membrane via the specific CAT-family proteins (cell cationic amino acid transporters) [7]. CAT-2B is usually induced under inflammatory conditions in a variety of cells. The upregulated CAT-2B is the main arginine transporter for activated macrophages, which import large amounts of extracellular arginine for iNOS-derived NO synthesis. It would be interesting to study any possible involvement of this protein in the mechanism of cell exasperation induced by oxidative stress, which if prolonged might lead to inflammation and ultimately cancer [8,9].

A fundamental mechanism in changing gene expression, reducing oxidative damage, and decreasing inflammation is represented by the use of natural compounds [10,11]. Over the past two decades, an increased number of researches have been focused on non-essential components such as phytochemicals of quinoa due to their potential capacity in reducing the inflammatory process beyond nutritional functions provided by its components [12]. 

*Chenopodium quinoa Wild* has attracted interest in the scientific community due to its nutritional features. It is a “pseudocereal” grain, a gluten-free food, rich in macronutrients such as carbohydrates, fats, and proteins of high nutritional quality for the correct balance of the essential amino acids, and micronutrients such as vitamins and minerals [13,14]. We have demonstrated that *Amaranthus retroflexus* L. belongs to the Amaranthaceae family and contains the oxyprenylated phenylpropanoids, a rare class of biologically active natural products as 7-isopentenyloxycoumarin, auraptene, umbelliprenin, and 4′-geranyloxyferulic acid (GOFA) [15]. As *C. quinoa* belongs to the same taxonomic family we also investigated the presence of oxyprenylated phenylpropanoids in the ethanol extract of *C. quinoa* seeds using commercially available roasted seeds (which is world-wide the most popular edible form of this plant). Recently, we have reported that the dietary administration of an inclusion complex of GOFA with β-cyclodextrin or its co-drug, with a known NOS inhibitor (L-NAME), effectively suppresses colitis-associated colorectal carcinogenesis in the AOM/DSS mouse model reducing the levels of inflammation mediators and inhibiting cell proliferation by inducing apoptosis [16,17]. However, the underlying mechanisms are still unclear. In this study we investigated the possible effects *C. quinoa* components might have in the modulation of oxidative stress and phlogosis in the human colon cancer cell line HCT 116 focusing on l-arginine-related signaling which can lead to cancer progression.

## 2. Results

The presence of oxyprenylated secondary metabolites was assessed using maceration for 96 h with EtOH. Using the gradient program already employed in the case of *A. retroflexus* [18], only GOFA was detected. The main retention times and other relevant parameters recorded for GOFA are summarized in Table 1. 

**Figure 1:**
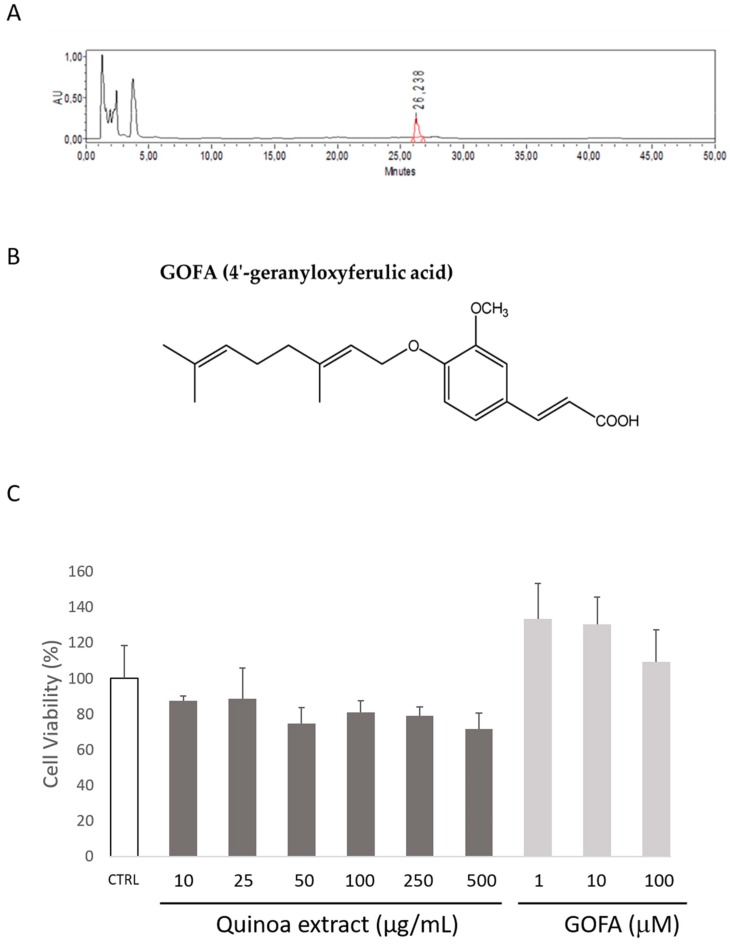
(**A**) HPLC chromatogram of quinoa roasted seeds ethanolic extract. Peak with Rt of 26.2 min identifies 4′-geranyloxyferulic acid (GOFA). (**B**) Chemical structure of GOFA. (**C**) The effects of the quinoa extract (10–500 µg/mL) and GOFA (1–100 µM) on HCT116 cell viability, measured by the MTT assay (24 h). Data are expressed as the mean ± SEM of at least three independent experiments, performed in triplicate.

Conditions set for column purge and re-equilibration ensured stability for column pressure and chromatogram background. The dead retention time, calculated with uracil, was 1.83 min. LOD was assessed to be 0.1 μg/mL. Calibration curves were linear over the range tested for pure analyte. The determination coefficient (r^2^) was ≥0.9991. Bias values ranged from –5% to 5.8%. The back-calculated concentration value, obtained from the calibration curves, allowed calculating 0.3 µL/mL as the validated LOQ. The weighting factor consisted of 1/x^2^ values. The mean imprecision values (CVs) of the retention time were 0.5% with no statistical differences compared with the inaccuracy for the normalized retentions time of the standard. Short-term stability of the analyte in vegetable matrix was studied under two experimental conditions: storage in an autosampler (20 °C) for 15 h and after three freeze–thaw cycles. A representative HPLC chromatogram of quinoa roasted seeds ethanolic extract is presented in Figure 1A. 

### 2.1. Quantification of GOFA in Roasted Quinoa Seeds Extracts

The recorded content of GOFA in quinoa seeds ethanol extract was 2.01 ± 0.08 mg/g of dry plant. Analytical data for this isolated phytochemical were in full agreement with those previously reported for the same compound [15]. Although we used several other solvents to accomplish the extraction process, the best experimental conditions leading to the highest yields in GOFA consisted of performing maceration with only EtOH. In all other cases, yields were by far lower. The ethanolic extract of quinoa roasted seeds was thus selected to carry out biological assays, and as a comparison with the activity displayed with pure GOFA. 

### 2.2. Quinoa and GOFA Effects on Cell Viability

First, HCT116 cell viability after treatment with various concentrations of the quinoa extract (10, 25, 50, 100, 250 and 500 µg/mL) and GOFA (1, 10 and 100 µM) was performed. As shown in Figure 1C, quinoa extracts did not show cytotoxic effects in any of the different concentrations applied. Similarly, the results of MTT test performed with the GOFA showed that the tested concentrations were not toxic to the cells (1, 10 and 100 µM).

### 2.3. Quinoa and GOFA Effects on Redox Balance

**Figure 2:**
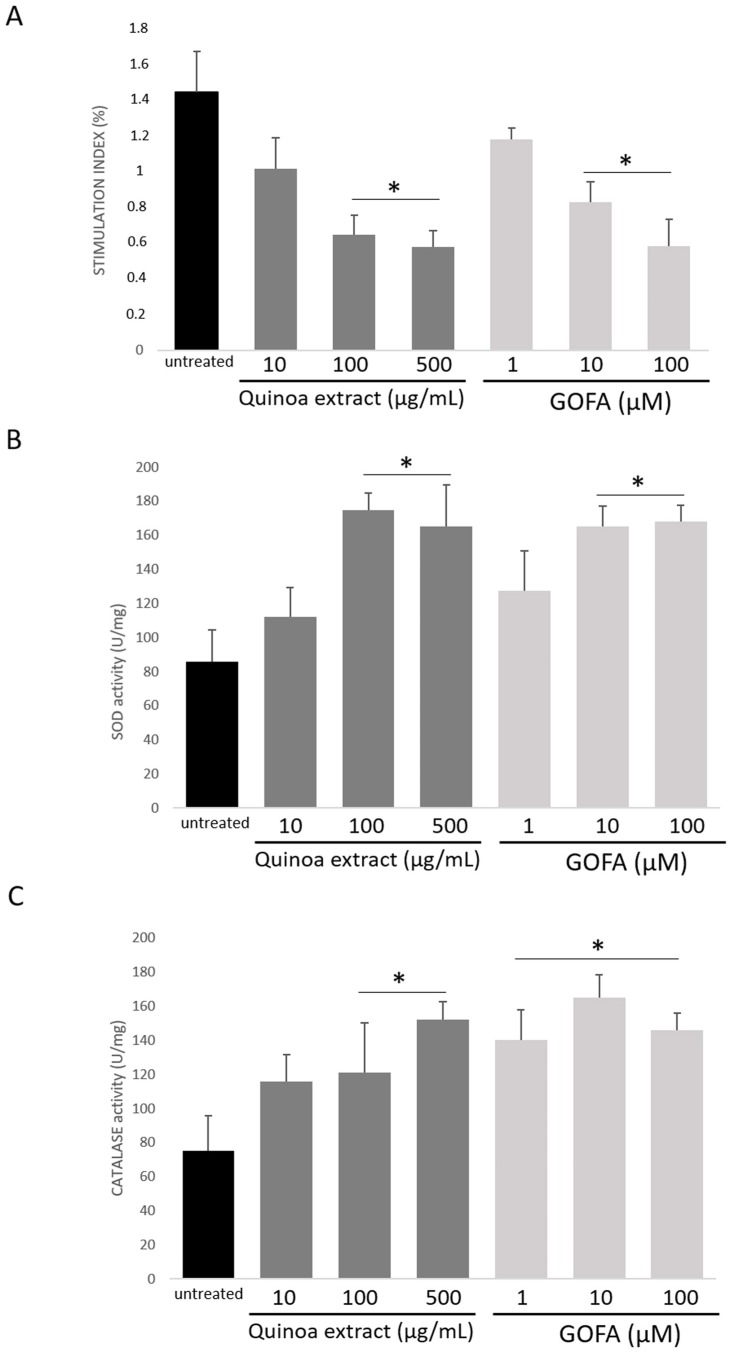
The effect of quinoa extract and GOFA on reactive oxygen species (ROS) production and anti-oxidative enzyme activity in HCT116 cells. (**A**) ROS production was measured by NBT assay. (**B**) Superoxide dismutase (SOD) and (**C**) catalase activity were analyzed. The results are representative of three different assays. Data are expressed as the mean ± SD. * *p* < 0.05 vs. untreated cells.

Inflammation leads to the production of reactive oxygen species (ROS), resulting in oxidative stress, activation of inflammatory response, and cellular damage [19,20]. The measure of ROS intracellular production represents a very useful parameter to quantify the ability of quinoa extract or GOFA in reducing oxidative stress. Therefore, the cytoprotective effects of quinoa extract and GOFA were also confirmed by NBT assay, in which the level of superoxide anion was measured. Quinoa extract and GOFA display an antioxidant effect on ROS production in a dose-dependent manner (Figure 2A). In HCT 116 cells, a significant reduction for ROS was obtained with a quinoa extract concentration of 100 μg/mL (*p* < 0.05). Instead, GOFA treatment showed a reduction of the ROS amount compared to the untreated cells, which became significant at 10 µM (*p* < 0.05). These preliminary data allowed obtaining a first observation of the concentration of quinoa extracts as well as GOFA, and these will be processed for further analysis. The doses of quinoa extract and GOFA used for all further analysis was 100 μg/mL and 10 µM respectively.

The ability of GOFA to attenuate the oxidative stress caused by inflammation in cancer cells was confirmed by the analysis to detect the antioxidant enzymes activity of superoxide dismutase (SOD) and catalase which is responsible for scavenging metabolites generated by free radicals, in cell homogenates. The untreated cells exhibited a reduction in the activity of antioxidant enzymes (*p* < 0.05, Figure 2B,C). Cells treatment with quinoa and GOFA promotes significant increase in the activity of these enzymes, 24 h post stimulation. However, the activity level of SOD in cells treated with quinoa and GOFA was significantly increased compared to the untreated cells (*p* < 0.05; Figure 2B). Regarding catalase, the treated cells showed a weak activity compared to the untreated cells (*p* < 0.01). Quinoa extract/GOFA-treatment resets the enzyme function which regained its normal metabolic and detoxifying function (*p* < 0.05; Figure 2C). Thus, quinoa and GOFA both act in restoring the cellular turnover of antioxidant enzymes of the HTC 116.

### 2.4. Quinoa and GOFA Effects on iNOS/NO/3-Nitrotyrosine

**Figure 3:**
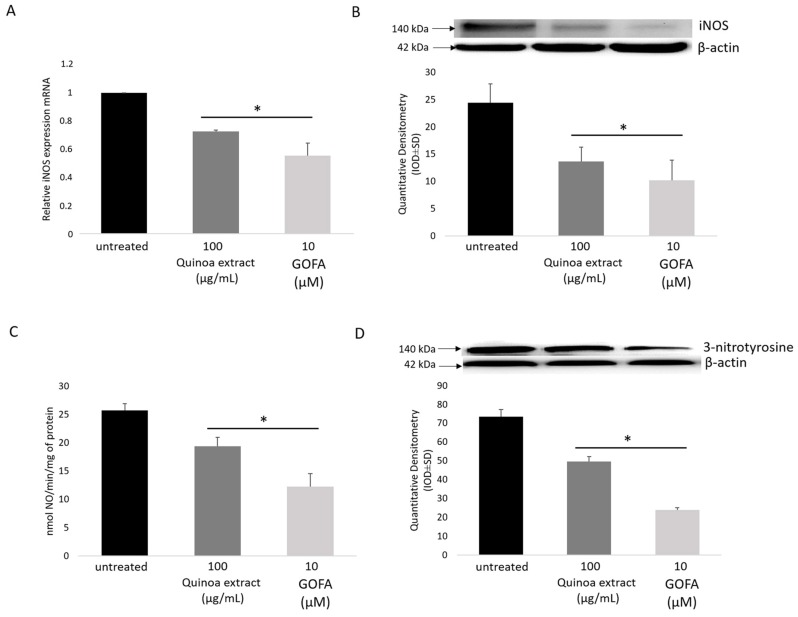
The effects of quinoa and GOFA on iNOS/NO/3-NitroTyrosine signaling in HCT-116 cells. The effects of quinoa extract and GOFA on: (**A**) iNOS mRNA levels, (**B**) iNOS protein expression. (**C**) NO production. (**D**) 3-nitrotyrosine protein expression. Each bar represents mean ± SD (*n* = 3, * *p* < 0.01 vs. untreated cells).

The expression of the inducible isoform of NOS and/or its catalytic activity increases in oxidative stress condition, as well as in inflammation. Thus, compounds that can selectively inhibit irregular expression of iNOS might be potential antioxidant and anti-inflammatory agents [21]. Our experimental study evidenced an elevated mRNA and protein expression of iNOS in HTC 116 cells (Figure 3A,B). In this context, it is interesting to note that real-time data and immunoblotting, point out that quinoa extract and GOFA induced a significant down regulation of iNOS. Moreover, NO production was also significantly decreased in quinoa and GOFA treated cells (Figure 3C). 

Generally, NO and O_2_^•−^, are observed at very low levels in the cellular microenvironment, when produced by constitutive enzymes. In altered redox state condition, if NO is produced by inducible isoform of nitric oxide synthase, it becomes highly reactive and might combine with O_2_^•−^ forming peroxynitrite (ONOO^−^), which is a strong oxidant and a nitrating agent. High levels of peroxynitrite induce nitrosative stress, which is due to the nitration of many amino acids, like tyrosine and tryptophan [21]. The level of nitrosylated proteins indirectly represents peroxynitrite levels in vitro. As expected, the 3-NT formation increased remarkably in the HCT116 cells as revealed by immunoblotting analysis of cell homogenates, which were attenuated by quinoa and GOFA pretreatment (Figure 3D).

### 2.5. Effects of GOFA on the Expression of Cationic Amino Acid Transporters in HCT-116 Cells

**Figure 4:**
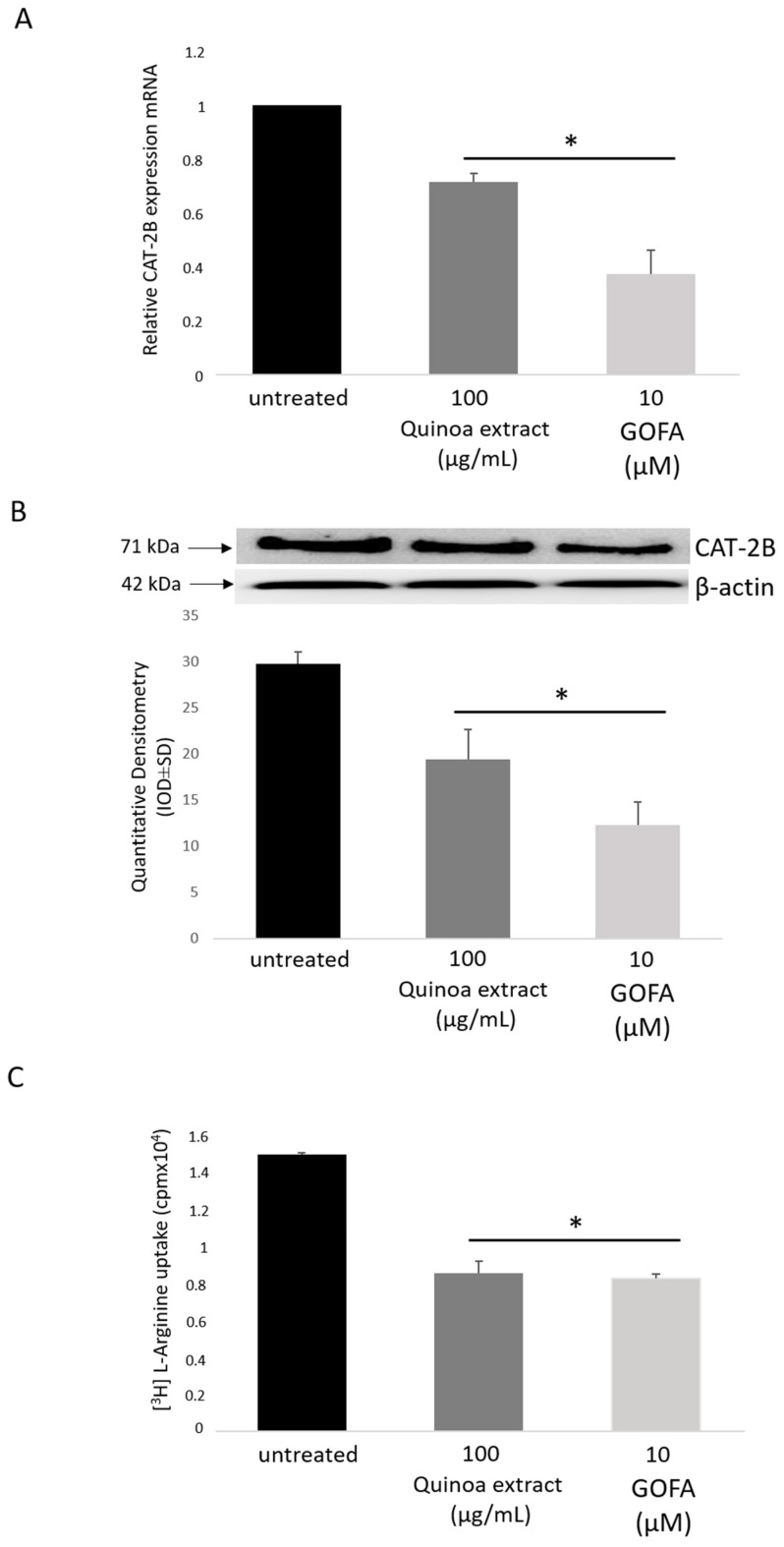
The effect of quinoa extract and GOFA on l-arginine transport in HCT116 cells. (**A**) mRNA expression of the CAT-2B. (**B**) Representative Western blot and densitometric analysis of CAT-2B expression. (**C**) (^3^H) l-arginine uptake. Data are expressed as the mean ± SD. * *p* < 0.05 vs. untreated cells.

The formation of NO depends not only on the activity of iNOS, but also on the availability of its substrate arginine (Arg). As stated above, arginine is taken up into cells by cationic amino acid transporter (CAT) systems in the plasma membrane. To test this, we first evaluated the mRNA expression of the l-Arg transporter, CAT-2B, in HCT 116 cells with the presence or absence of quinoa extract or GOFA. As reported in Figure 4A,B, the addition of quinoa extract or GOFA decreased CAT-2B expression suggesting that l-Arg uptake via CAT-2B was modified in quinoa extract/GOFA-treated cells. Subsequently, to evaluate the functional activity of CAT-2B, we directly measured l-Arg uptake through radioactivity incorporation in HTC-116 cells (Figure 4C). l-Arg uptake was significantly greater in untreated cells. In cells treated with quinoa extract or GOFA, the concentration of intracellular l-Arg was decreased comparing to untreated cells.

### 2.6. Quinoa Extract and GOFA Effects on the Pro-Inflammatory Markers

**Figure 5:**
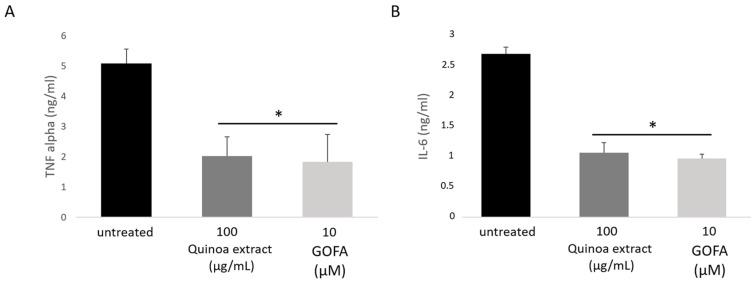
The effect of quinoa extract and GOFA on TNF-α and IL-6 production in HCT-116 cells. TNF-α (**A**) and IL-6 (**B**) levels were measured by ELISA. Values are means standard deviations; *n* = 3, * *p* < 0.05 vs. untreated cells.

Given that ROS overproduction and antioxidant enzyme activities reduction play an important role in the inflammatory response in cancer cells, we measured the levels of cytokines as TNF-α and IL-6 within HCT 116 cells using ELISA test (Figure 5). Cells were treated with QUINOA 100 µg/mL and GOFA 10 µM for a period of 24 h. We observed that TNF-α and IL-6 increased in untreated cells. The Quinoa extract and GOFA attenuated the level of pro-inflammatory cytokines studied (TNF-α and IL-6). 

### 2.7. Effect of CAT-2B Silencing on the l-Arg/iNOS Pathways Levels

**Figure 6:**
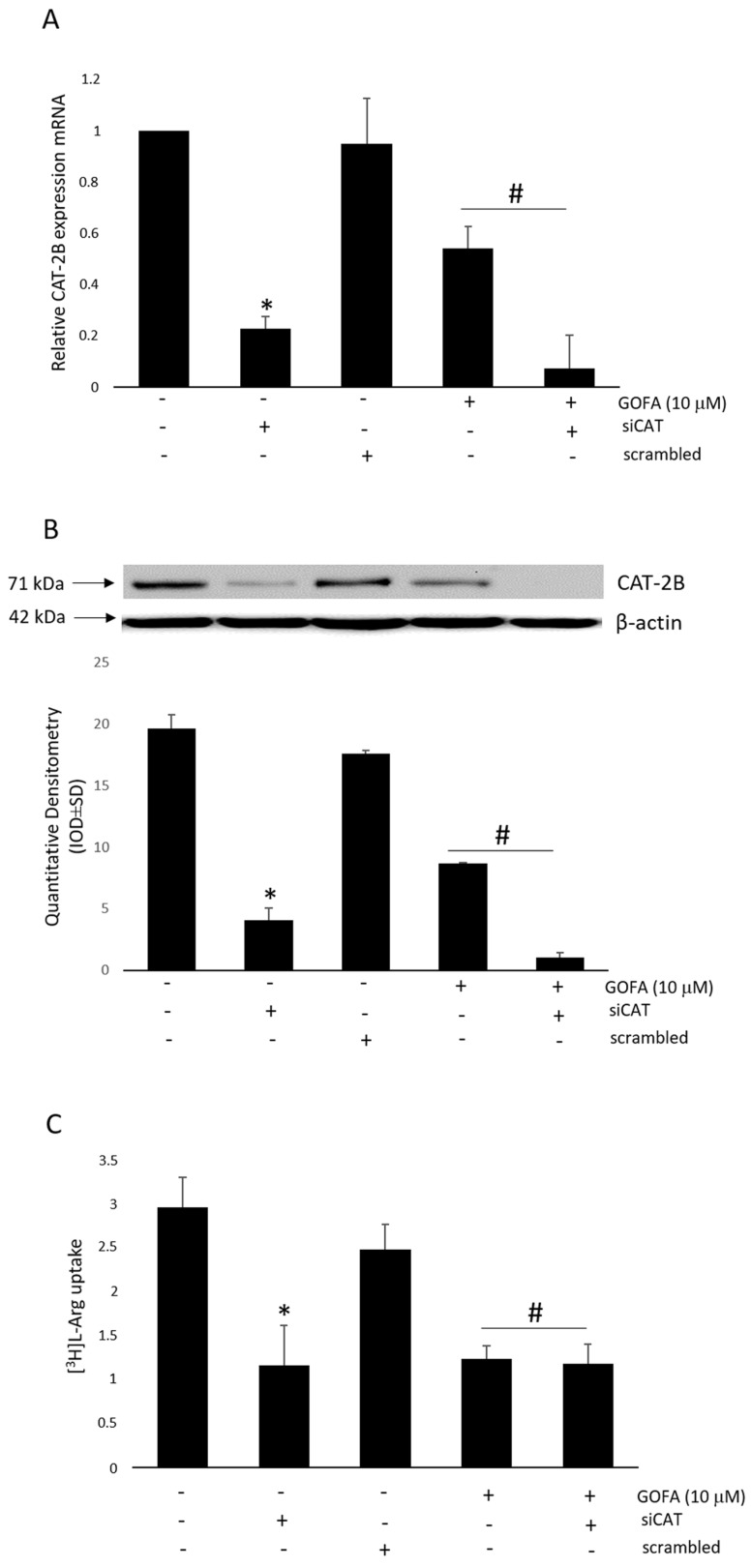
The effect of quinoa extract and GOFA on CAT2B expression and l-arginine uptake in HTC116 transfected with CAT2 siRNA. (**A**) mRNA expression of the CAT2B. (**B**) Representative Western blot and densitometric analysis of CAT 2B expression. (**C**) (^3^H) l-Arg uptake by HCT116 cells. Data are expressed as the mean ± SD. * *p* < 0.01 vs. untreated cells; # *p* < 0.01 vs. relative control cells not treated with GOFA.

To assess whether the GOFA regulates iNOS by interfering with the intracellular concentration of l-arginine in HCT116 cells, the transporter CAT-2B was silenced with a siRNA specific for the coding sequence. Cells were transfected with scrambled or CAT-2B siRNA. Data presented in Figure 6A show the expression of CAT-2B transporter, evident in untreated cells, in comparison to cells transfected with CAT-2B siRNA in which it was abolished. The observed changes of gene expression were associated with changes in protein levels (Figure 6B). Furthermore, the cellular uptake of arginine decreased in cells silenced for CAT-2B compared to untreated ones (Figure 6C). 

**Figure 7:**
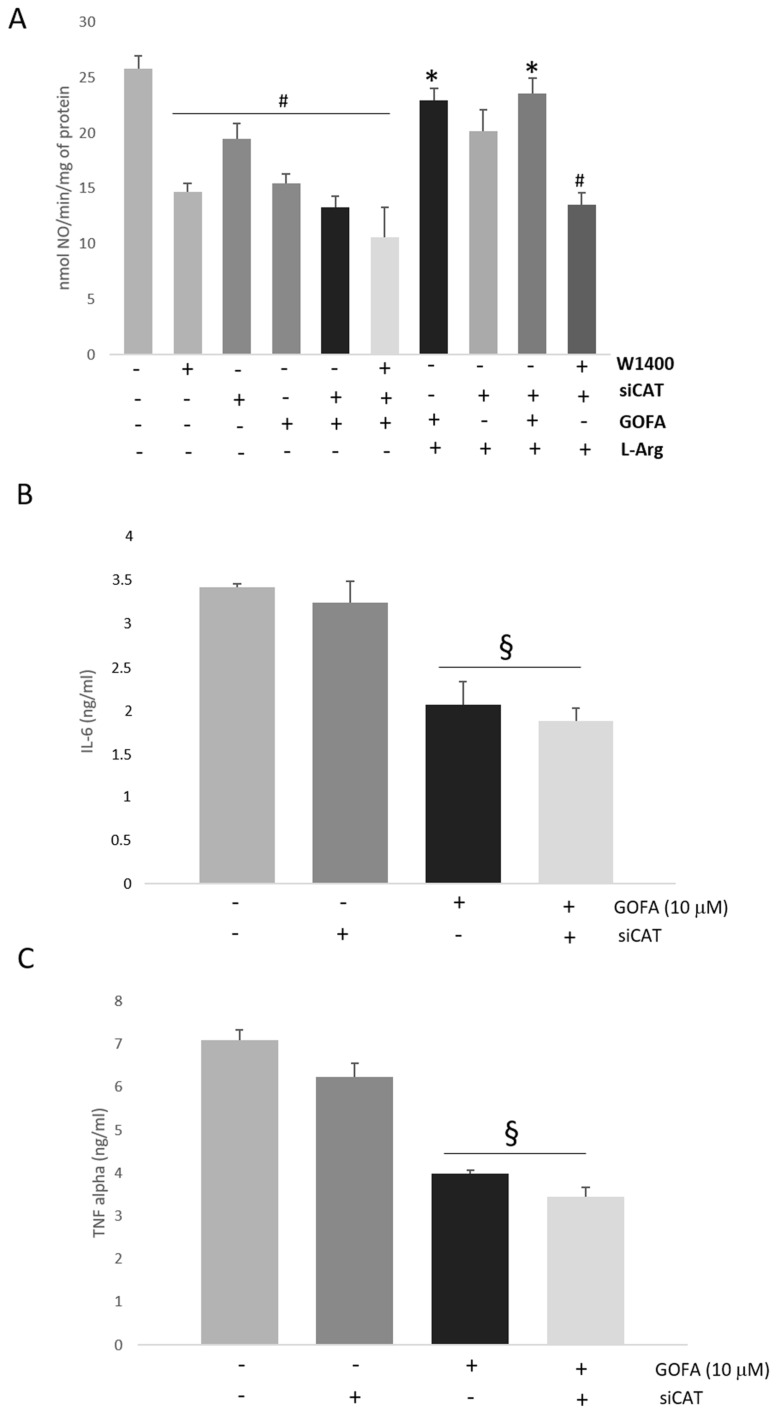
(**A**) The effect of W1400, siCAT and l-arginine on iNOS activity in HCT 116 cells. The iNOS activity was measured by oxyemoglobin assay (*n* = 4). (**B**) The effect of CAT2 silencing on IL-6 production. (**C**) The effect of CAT2 silencing on TNF-α production. Data are expressed as mean ± SD. # *p* < 0.05 vs. untreated cells; * *p* < 0.05 vs. cells not treated with l-Arg. ^§^ *p* < 0.05 vs. relative control cells not treated with GOFA.

In order to verify whether GOFA acts as CAT-2B repressor in eliciting l-Arg uptake in HCT116 cells, we performed iNOS activity assay experiments (Figure 7A). First, the GOFA induced NO to decrease and it also decreased its selectivity towards the inducible isoform; this was confirmed by comparing it to the selective iNOS inhibitor, the W1400. When the cells were treated with a specific siCAT-2B we noted a reduction of iNOS activity compared to untreated cells, confirming that iNOS activation was dependent on CAT-2B inducted l-arginine uptake. In cells with poor expression of CAT-2B, treated with GOFA, we did not observe a significant change in iNOS activity compared to the cells treated only with GOFA. This data was confirmed by co-incubation, in silenced cells, with both the W1400 and GOFA. The data reported in Figure 7A suggest that GOFA effects are mediated by its ability to downregulate the expression of inducible CAT-2B transporter. To further confirm our hypothesis, cells were supplied with l-arginine 100 μM, a concentration that can induce iNOS activity in untreated cells. The dose-response investigation showed that the levels of NO were significantly elevated. It is important to note that, in HTC116 cells with high enough levels of l-arginine and elevate expression of CAT-2B, the treatment with W1400 significantly reduced iNOS activity. We did not observe any variations in GOFA treated cells under the same experimental conditions.

Finally, the expression of pro-inflammatory cytokines, such as IL-6 and TNF-α, was not affected by the expression of CAT-2B, as our genes were induced alike both in untreated and CAT-2B-silenced cells (Figure 7B,C). Our results suggest that GOFA inhibits iNOS activity through downregulation of CAT-2B.

## 3. Discussion

Colorectal cancer (CRC), a malignant tumor of the large intestine, is the third most common cancer in the world. The difficulty of understanding CRC is largely attributable to multifactorial etiology [22].

Yet, as research progresses, some factors that seem to be the basis of many types of tumors, such as inflammation, appear to be very clear, and their link with cancer, especially regarding the acquisition of malignancy, appears to be a crucial element [23,24].

Specific oxidative processes in the tumor microenvironment closely link inflammation and cancer. Therefore, oxidative enzymes, known to play a key role in inflammation, are increasingly investigated and linked to cancer as CRC [24,25]. The interest in natural compound-based medicines as a remedy for inflammation has increased in recent years. The natural compounds from plant extracts are the subject of scientific interest because of their function in the progression of disease [26,27,28]. Recently C. *quinoa* has attracted the attention of the scientific community for its effects on human health, and even today many of its properties are unknown [29]. Thus, we decided to investigate the presence of new biologically active O-prenylated phenylpropanoids in different extracts of commercially available quinoa seeds. These natural compounds are already known to possess noteworthy biological effects and great therapeutic potentialities as chemo-preventive, anti-inflammatory, neuroprotective, and anti-microbial agents [30]. Only GOFA was detected as the major O-prenylated phenylpropanoid phytochemical in ethanolic extracts from quinoa seed (Figure 1A).

As stated above, GOFA is a prenyloxycinnamic acid which has been seen to exert a remarkable antitumor effect, and since the inflammation plays a key role in the progression of tumor pathology, we investigated the protective effects of quinoa extract and GOFA against inflammation and oxidative stress in human colon cancer cell line HCT-116. Our attention focused on the study of different signaling and molecular pathways NO-dependent. The HCT-116 cells exhibit an altered redox homeostasis, characterized by high levels of superoxide radical due to the impairment of the intracellular antioxidant network of SOD and catalase (Figure 2). Nitric oxide (NO) is a critical pro-inflammatory mediator in triggering inflammatory response implicated in the progression of cancer [31]. HTC-116 cells were characterized by an upregulation of inducible nitric oxide synthases, (iNOS) expression, and a large amount of NO (Figure 3). Generally, when the NO is produced in large quantities, it reacts with oxygen radicals forming peroxynitrite, which are strong oxidizing and nitrous agents, resulting in nitrosylation of cellular signaling proteins. Untreated HCT116 cells show high levels of 3-NT (Figure 3C) which play a role in the pathogenesis of several diseases inducing an irreversible form of protein damage. We have therefore studied the ability of quinoa extract and its main component, GOFA, to reduce cellular redox imbalance and over-production of pro-inflammatory molecules. Our data evidenced that quinoa extract and GOFA counteract the oxidative stress and restore the redox balance by activation of defensive antioxidant enzymes.

In HCT116 cells both quinoa extract and GOFA, blocked NO production. This leads to a decrease in the formation of ONOO^−^ which ultimately lead to the downregulation of iNOS. Many studies have reported that l-arginine (l-Arg) plays an important role in the intestinal physiology such as cell growth, proliferation, migration, and protein synthesis [32,33]. In the cell, l-Arg is metabolized by arginases to l-ornithine in the polyamine biosynthesis, and by nitric oxide synthases (NOS) to l-citrulline and NO. The cationic amino acid transporter (CAT) mediates the cellular uptake of l-Arg [8]. Since in our cells treated with GOFA we found a significant reduction in the activity of the inducible isoform of NOS, we evaluated the uptake of its substrate, l-Arg. As shown in Figure 4C, it is evident how the levels of l-arginine significantly decrease in cells treated with GOFA compared to untreated cells. For a better understanding of our results, we performed an expression study of the CAT-2B transporter, noting that it was significantly reduced in HCT116 cells treated with GOFA (Figure 4B). Therefore, with the results obtained, we hypothesize that GOFA in an altered redox balance has the ability to modulate a cellular response through down regulation of the iNOS/CAT-2B/l-Arg pathways. This hypothesis was confirmed through the silencing with a siRNA specific for the coding sequence of CAT 2 gene. As hypothesized, CAT2B silenced cells submitted to saturating concentrations of l-arginine and treated with GOFA, showed an elevated iNOS activity unlike the ones treated under the same experimental conditions, with 1400W, a selective iNOS inhibitor (Figure 7A). Therefore, we demonstrate that, in human cancer colon cell the GOFA, interfering with the expression of the CAT-2B transporter, decreases the uptake of l-arginine, acting as an indirect inhibitor of iNOS leading to a reduction of the activity of an inducible enzyme and therefore to a correlated decrease in NO. 

Since NO is an inflammatory mediator capable of modifying the inflammatory microenvironment, it is clear that its reduction mediated by the GOFA, can, in turn, lead to the modulation of different molecules involved in the process. For this reason, and since recent studies suggest the existence of a network of NO-linked regulatory cytokines implicated in the progression of chronic inflammation like in CRC, we wanted to measure the levels of inflammatory cytokines such as TNF-alpha and IL6. Our results indicate that GOFA expresses the ability of attenuating the level of pro-inflammatory cytokines studied. In this study, we were able to identify a new molecular target through which the GOFA modulates a transduction process due to the ability in downregulating the expression of the CAT-2B. 

In this study, we were able to identify a new molecular target through which the GOFA modulates a transduction process.

Our data, about l-Arg accumulation and overexpression of CAT-2B in HCT-116 cells displayed guaranteed further clarification of the role of l-Arg/iNOS/CAT-2B pathway in CRC cells and its biological importance in tumorigenesis. Moreover, our results lead us to promote the GOFA as a new candidate for the reduction of intestinal mucosal inflammation. In the future, it would be strongly desirable to increase the study of this potential therapeutic agent to assess its efficacy and safety.

## 4. Materials and Methods

### 4.1. Materials

Quinoa seeds were purchased from a local market. A voucher specimen (QS-2017-1) was stored in the deposit of the laboratory of Chemistry of Natural Compounds at the Department of Pharmacy of the University “G. D’Annunzio” of Chieti-Pescara. As already reported, GOFA was synthesized and its purity (>98.1%) assessed by HPLC and 1H NMR [34]. Methanol (HPLC grade), acetonitrile (HPLC grade), and acetic acid were purchased from Carlo Erba Reagents (Milan, Italy). HPLC-grade water (>18 MΩ/cm resistivity) was obtained by passage through an Elix 3 and Milli-Q academic water purification system (Millipore, Bedford, MA, USA). DMSO, fetal calf serum, streptomycin, penicillin, L-glutamine, 3-(4,5-dimethylthiazol-2-yl)-2,5-diphenyltetrazolium bromide, Nitro Blue-tetrazolium, catalase, xanthine, xanthine oxidase, Griess reagent, formic acid, 1400W (iNOS inhibitor), acetonitrile and methanol were obtained from Sigma-Aldrich (Merck KGaA, Darmstadt, Germany). The primary antibodies anti-iNOS, 3-nitrotyrosine were obtained from Santa Cruz Biotechnology (Santa Cruz, CA, USA). The primary antibodies anti-CAT2B were purchased from Thermo Fisher Scientific (Waltham, MA, USA). The secondary antibodies actin were obtained from Sigma-Aldrich (US/Canada). The colorectal carcinoma cell line HCT 116 were purchased from American Type Culture Collection (Manassas, VA, USA) and McCoy’s 5a medium was obtained from GIBCO (Thermo Fisher Scientific, Rodano, Italy).

### 4.2. Extraction Procedures and HPLC Analysis

Seeds were finely triturated by an Ultra-Turrax^®^ disperser prior to extraction. A sample of 1 g of seeds was extracted by maceration with 10 mL of EtOH. Analysis was performed using a Waters 600 HPLC system equipped with a Waters 2996 PDA detector, a Rheodyne manual syringe-loading valve injector model 7125 (Cotati, CA, USA) fitted with a 20 μL loop. Data acquisition was monitored by Waters Empower software (ver. 2.0). Chromatographic separation was achieved employing a GraceSmart RP18 (5 µm particle size, 250 mm × 4.6 mm, Grace, Deerfield, IL, USA). Column temperature was maintained at 25 ± 1 °C using a cool pocket chiller (ThermoScientific, Waltham, MA, USA). The detection was set at 322 nm. Elution mixture consisted of H_2_O and acetonitrile both combined with acetic acid (0.03%) (eluent A and eluent B respectively). The mobile phase was directly on-line degassed by using Infinity Agilent model 1260 (Agilent Technologies, Santa Clara, CA, USA). The flow rate was 1.20 mL/min. Chromatographic separation was carried out using the gradient elution already reported [34]. Method validation was achieved following the “Guidance for Industry-Bioanalytical Method Validation” recommended by the Food and Drug Administration (FDA) of the United States. Stock solutions for calibration curves were prepared by dissolving 10 mg of GOFA into 10 mL of MeOH and stored in glass-stoppered bottles at 4 °C. Standard for calibration curves and quality control samples (QC), at concentrations of 1.0, 10.0, 20.0, 30.0, 40.0, 50.0, 60.0, 70.0, 80.0, 90.0 and 100.0 μg/mL, were daily prepared by appropriate dilution aliquots of the stock solutions in MeOH. Pooled quality control samples of the analyte were prepared to determine the limit of quantification (LOQ), the intra- and inter-assay precision and accuracy of the method, and to evaluate the stability of compounds when stored under different conditions. QC samples at three different concentration levels (QClow = 5.0, QCmedium = 45.0, and QChigh = 95.0 μg/mL) were used to confirm the analytical run. On five separate days, six calibration curves were plotted against the corresponding concentrations. The calibration curve was also evaluated by its correlation coefficient, slope, and intercept. The limit of detection (LOD) was calculated from the calibration graphic based on the guidelines given by IUPAC and was defined as three times the standard deviation of a blank sample divided by the analytical sensitivity. The LOQ was defined as the lowest concentration on the calibration curve, which could measure (*n* = 5) with a precision (RSD%) not exceeding 20% and with an accuracy between 80% and 120%. The data for intra- and inter-day precision and accuracy were obtained from the analysis of three batches of LOQ and the QC samples at three different levels of analyte in duplicate on the same day and for five consecutive days according to international guidelines. The carry over was investigated on the same column used for the analysis by injecting blank vegetable sample extracted with EtOH followed by blank vegetable sample spiked with the analytes at the LOQ concentration into the HPLC system, followed by at least three blank vegetable extracts. No significant carry over effect (<0.43%) was recorded.

### 4.3. Cell Culture, Assay for Cell Viability and Cytotoxicity

#### 4.3.1. Cell Culture

The HCT 116 cells were cultured in a 5% CO_2_ atmosphere in McCoy’s 5a medium containing 10% fetal calf serum, 100 ng/mL streptomycin, 100 U/mL penicillin, and 2 mM L-glutamine. Cells were seeded (at 2 × 10^5^ cells per well) onto six-well tissue culture plates and cultured in medium with LPS (10 µg/mL) for 24h, in the presence or absence of the quinoa roasted seeds ethanol extract, GOFA. Control cells did not contain quinoa extract or GOFA. In all experiments, equal volumes of PBS or DMSO were added to the medium of control cultures (controls were performed using non-stimulated cells). The concentrations of l-arginine were chosen according to our preliminary optimization studies. After incubation, cells were harvested by centrifugation to assess cellular viability, gene and protein expression. Media of HCT 116 cells were collected in order to evaluate the NO, O_2_^−^ and cytokines release. For the experiments we used three different plates with three replicates in three different passages of cells, and each biological replicate was analyzed in triplicate in the experiments performed.

#### 4.3.2. MTT

To assess cell damage and cell viability by the quinoa extract and GOFA, the MTT assay was used. As reported previously, 2 × 10^4^ cells were seeded on 96-well plates, cultured and treated with quinoa extract or GOFA. To highlight the non-cytotoxic effect of quinoa and GOFA, LPS concentration was used as a positive control of cytotoxicity. The MTT reagent (20 µL) was added after medium (200 µL) to each well at a concentration of 0.5 mg/mL. After incubation at 37 °C for 4 h, in order to dissolve the formazan that had formed, the solution (220 µL) was removed from each well and DMSO (150 µL) was added. The absorbance of the formazan solution is read spectrophotometrically at 570 nm on an ELISA reader (Bio-Rad, Hercules, CA, USA). The following equation was applied to calculate the percentage of cell viability:

$$\%=\;\frac{Absorbance\;of\;treated\;cells}{Absorbance\;of\;control\;cells}\times 100$$

#### 4.3.3. NitroBlue-Tetrazolium Assay

Superoxide anion was detected with nitroblue tetrazolium (NBT). As described previously [20], to each well of a 96-well plate the following reagents were added: potassium phosphate buffer (100 μL, pH 7.8, 50 mM), catalase (5 μL), NBT (25 μM, 5.6 × 10^−9^ M), xanthine (50 μL, 0.1 mM), xanthine oxidase (50 μL, 0.1 mM), quinoa extract (concentration range between 10 and 500 µg/mL), and GOFA (1, 10, and 100 μM). NBT stock solution was prepared in distilled water and kept at 4 °C in the dark. From this stock solution, a NBT working solution was prepared in culture medium just before utilization. 

For the analysis 5 × 10^5^ cells were deposited in triplicate. Following the addition of NBT the plates were incubated for 1 h at room temperature until the blue color had developed and the absorbance was measured at 560 nm.

### 4.4. Antioxidant Enzymes Activity

#### 4.4.1. Superoxide Dismutase Activity Assay

Superoxide dismutase (SOD) (EC 1.15.1.1) activity was determined by using the superoxide anion to inhibit epinephrine autoxidation. The assay mixture contained: sodium carbonate buffer (50 mM, at pH 10), epinephrine (0.1 mM), and fresh cell lysate (containing 25 μg of protein) in a final volume of 2.5 mL. To differentiate the CN-insensitive MnSOD from the CN-sensitive Cu, ZnSOD solution of the KCN (1.25 mM) was employed. The capability of the SOD to inhibit the auto-oxidation of epinephrine was monitored spectrophotometrically at 480 nm at 25 °C. Percentage inhibition values were converted into activities by using a purified Cu, Zn bovine SOD as standard. One unit of SOD is the amount of enzyme required to halve the rate of substrate auto-oxidation.

#### 4.4.2. Catalase Activity

Catalase (CAT) (EC 1.11.1.6) activity was measured spectrophotometrically at 240 nm monitoring the decomposition of H_2_O_2_. 1.5–11 μg of protein of enzymatic extract was added to the assay mixture containing potassium phosphate buffer (50 mM, pH 7) and H_2_O_2_ (10 mM). The A_240_ of this solution in a final volume of 2 mL was monitored using Phosphate Buffer as a blank. CAT units were defined as 1 μmole H_2_O_2_ decomposed/min at 25 °C.

### 4.5. Expression Analysis

#### 4.5.1. Quantitative Real-Time PCR

Real-time PCR RNA expression assay was carried out in an Eppendorf Mastercycler EP Realplex (Eppendorf AG) and the amplifications were done using the SYBR Green PCR Master Mix (Applied Biosystems) [35]. Primers and GAPDH as control were designed using GeneWorks software (IntelliGenetix, Inc., Mountain View, CA, USA). The primer pairs used were as follows: iNOS forward- 5′-TTCAGTATCACAACCTCAGCAAG-3 and reverse 5′-TGGACCTGCAAGTTAAAATCCC-3′; CAT-2B-forward 5′-GACCTTTGCCCGATGTCTGAT-3′ and reverse-5′-AGCAGCGGCATAATTTGGTGT-3′; GADPH forward -5′-GAAGGTGAAGGTCGGAGTC-3′ and reverse -5′-GAAGATGGTGATGGGATTTC-3′. DNA was denatured at 95 °C for 10 min. followed by 40 cycles of melting for 30 sec. at 95 °C together with annealing/extension for 60 sec. at 60 °C. The relative quantification in gene expression was determined using the 2^−ΔΔCt^ method. Data are representative of three different experiments each run in triplicate, and are presented as the mean ± SEM of triplicates. The experiments were repeated twice with consistent results.

#### 4.5.2. Western Blot Analysis

For the preparation of total protein extracts, 2 × 10^5^ cells/mL were seeded in a six-well plate, cells were washed twice with PBS and lysed in lysis buffer (RIPA). The proteins were quantified using the Bradford Method. The Western blot analysis was performed as described previously [36]. Briefly, the lysates were electrophoresed on 12% SDS-PAGE and then electrotransfered to a nitrocellulose membrane. The primary antibodies [anti-iNOS, anti-3-nitrotyrosine and anti-CAT-2B (dilution 1:500) and *β*-actin (dilution 1:5000) were incubated at 4 °C overnight. 

The membranes were washed three times with PBS-Tween20 (0.1%) in for 20 min, followed by reacting with HRP-conjugated goat anti-rabbit IgG and HRP-conjugated rabbit anti-mouse IgG (for *β*-actin) at RT for 2 h. Then, the membrane was washed three times (20 min each wash) with PBS-Tween20 (0.1%). The nitrocellulose was scanned using the Bio-Rad Gel Doc 1000 and the Quantity One 4.4.1 software. Saturated images of technical replicates were discarded.

### 4.6. Analysis of the l-Arg/iNOS/NO System

#### 4.6.1. NOS Activity

To evaluate the NOS activity, oxyhemoglobin assay was performed. iNOS activity was assessed in calcium-free conditions using an assay mixture contained l-arginine (10 μM), NADPH (100 μM), tetrahydrobiopterin (6.5 μM), and oxyhemoglobin (3 mM) in HEPES (100 mM, pH 7.5). The assays were initiated with lysate addition (40 μg of protein) in a final volume of 200 µL. Nitric oxide reacts with oxyhemoglobin to yield methemoglobin which is detected at 576 nm (*e* = 12,000 M^−1^cm^−1^) on a Perkin-Elmer LamdaBIO UV–vis spectrophotometer.

#### 4.6.2. Measurement of NO Release

To detect the NO release from cells, 2 × 10^6^ cells were seeded in six-well plate and nitrite was measured in culture supernatants. The assay was performed as described previously [37]. Sodium Nitrite, at concentrations of 0 to 100 μM, was used as a standard to assess nitrite concentrations. Aliquots of the culture supernatant were mixed with an equal volume of the Griess reagent and absorbance was determined at 540 nm using a microplate reader. 

#### 4.6.3. l-Arg Uptake Assay

(^3^H) l-arginine was purchased from PerkinElmer, Singapore. Triplicate HCT116 cultures (5 × 10^6^ cells resuspended in 2 mL of serum free culture medium lacking l-Arg) were treated with quinoa and GOFA. Eighteen hours later, plates were washed with warm (37 °C) PBS and then incubated with 0.1 mM (^3^H) l-Arg (Perkin Elmer, Boston, MA, USA) in warm PBS for 2 min. Plates were then washed twice with warm PBS, and cells were lysed in RIPA buffer. Cell lysate were mixed with scintillation fluid and radioactive cpm were measured. Arginine transport was expressed as (^3^H) l-Arg uptake (cpm × 10^4^). 

### 4.7. siRNA Transfection

HCT116 cells (1 × 10^6^) were seeded in a six-well plate. Fresh siRNA transfection reagent was prepared diluting first Lipofectamine RNAiMAX in Opti-MEM for 5 min and then mixed with an equal volume of Opti-MEM containing the siRNA (final concentration 25 nM). After 20 min of incubation, 200 µL of the resulting RNAiMAX/siRNA was added directly onto the cells and cultured for 6 h. Then, the supernatant was removed and cells were further cultured with fresh complete growth medium for 12–1 h before any treatment with 1400W (50 µM), l-arginine (150 µM) and GOFA. qRT-PCR was used to confirm siRNA-mediated downregulation of the target gene. Control cells were without siRNA. Cell death was detected using MTT, showing no reduction of cell viability. siRNA for CAT-2 (sc-77441) was purchased from Santa Cruz Biotechnology. OptiMEM and Lipofectamine RNAiMax reagents were purchased from Invitrogen Life Technologies, USA.

### 4.8. Cytokine’s Levels Measurement

Briefly, HCT-116 cells were pretreated with GOFA (10 µM) and Quinoa (100 mg/mL) for 24 h. The supernatants were collected and assayed using the Searchligth Elisa kit according to the manufacturer’s instructions (Thermo Fisher Scientific, Rockford, IL, USA).

### 4.9. Statistical Analysis

All results were expressed as mean ± SD or SEM from three independent experiments. For statistical analyses, quantitative data were analyzed by Student t test for unpaired data between treated to non-treated cells. A probability of null hypothesis of <5% (*p* < 0.05) was considered as statistically significant.

## Figures and Tables

**Table 1:** Mean linear calibration curve parameters obtained by weighted linear least-squares regression analysis of three independent twelve non-zero concentration point.

Entry	Linearity Range (μg/mL)	Calibration Curve	Weighting Factor	Determination Coefficient (r^2^)	Rt (min) *
GOFA	1–100	Y = 24142x + 3054	1/x^2^	0.9993	41.77

* Rt = Retention time.
